# Supplementary material for: Characterisation of facial expressions and behaviours of horses in response to positive and negative emotional anticipation using network analysis
Source: PLoS One. 2025 May 14;20(5):e0319315. doi: 10.1371/journal.pone.0319315 (PMC12077714; doi:10.1371/journal.pone.0319315)
Supplement: S1 Table — (PDF) [file pone.0319315.s001.pdf]

S1 Table. Results of inter-coder reliability (Intra-class correlation coefficient test and Cohen's Kappa test).

Variable	Type of test	Result	Level of agreement
AD1	ICC	0.53	Moderate
AD19	ICC	1	Excellent
AD38	ICC	0.88	Excellent
AD81	ICC	0.97	Excellent
AD133	ICC	0	Poor
AU5	ICC	0.53	Moderate
AU16	ICC	0.37	Poor
AU17	ICC	0.90	Excellent
AU101	ICC	0.54	Moderate
AU113	ICC	0.95	Excellent
AU145	ICC	0.92	Excellent
AU47	ICC	0.83	Excellent
AUH13	ICC	1	Excellent
Ears position	Cohen's Kappa	0.74	substantial
Neck position	ICC	0.94	Excellent
AD50	ICC	1	Excellent
AD84	ICC	0.8	Excellent
AD85	ICC	0.49	Poor
Paw the Ground	ICC	0.99	Excellent
Observe Congener/ Experimenter	ICC	0.99	Excellent
Step Back	ICC	1	Excellent
Step Further	ICC	1	Excellent
Sniff the Ground	ICC	0.99	Excellent
