# Supplementary material for: Characterisation of facial expressions and behaviours of horses in response to positive and negative emotional anticipation using network analysis
Source: PLoS One. 2025 May 14;20(5):e0319315. doi: 10.1371/journal.pone.0319315 (PMC12077714; doi:10.1371/journal.pone.0319315)
Supplement: Attachment — Submitted filename: Response to Reviewers.pdf [file pone.0319315.s004.pdf]

Point-by-Point Response to Reviewers' Comments

PONE-D-25-05198

Characterisation of facial expressions and behaviours of horses in response to positive and negative emotional anticipation using network analysis
PLOS ONE

All suggested corrections have been made and are detailed below (in blue), with references to the corresponding line numbers in the document 'Revised Manuscript with Track Changes', in Revision mode: All Markup and Show Markup / Balloons / Show All Revisions Inline.

We would like to sincerely thank the reviewers for their constructive and highly relevant comments on the manuscript, which have greatly contributed to its improvement and enhanced the quality of our work.

Journal Requirements:

- When submitting your revision, we need you to address these additional requirements.

1. Please ensure that your manuscript meets PLOS ONE's style requirements, including those for file naming. The PLOS ONE style templates can be found at https://journals.plos.org/plosone/s/file?id=wjVg/PLOSOne_formatting_sample_main_body.pdf https://journals.plos.org/plosone/s/file?id=ba62/PLOSOne_formatting_sample_title_authors_affiliations.pdf

We have reviewed and revised the manuscript to comply with PLOS ONE's style requirements.

In the title page:

- We have formatted the title in regular (non-bold) text (L1-L2).
- We have adjusted the affiliations according to the instructions provided in the template (L7, L8, L9-L10).
- We have formatted the corresponding authors in accordance with the template guidelines (L13).

In the manuscript:

- Font: Times New Roman.
- Paragraph indentation has been applied.
- Double spacing has been applied also for the Tables.
- The format of the Tables has been modified in accordance with the instructions and template provided.
- References have been formatted in square brackets, following the PLOS ONE style.
- Line 189: citation corrected to '(Fig 2A)' instead of '(Fig 2 A)'.
- Line 191: citation corrected to '(Fig 2B)' instead of '(Fig 2 B)'.
- Mentions of funding have been removed from the acknowledgments section (L739-L742).
- The 'Supporting Information' section at the end of the manuscript has been correctly renamed (L967).

- 'Table S1' has been renamed to 'S1 Table' (L968) and 'Table S2' to 'S2 Table' (L970) in the Supplementary Information and throughout the manuscript.

For file naming:

- The Supplementary Information files have been correctly renamed (‘S1_Table.pdf’ and ‘S2_Table.pdf’) and uploaded as individual files.

2. We note that the grant information you provided in the ‘Funding Information’ and ‘Financial Disclosure’ sections do not match.

When you resubmit, please ensure that you provide the correct grant numbers for the awards you received for your study in the ‘Funding Information’ section.

During the resubmission phase, we will ensure that the correct and consistent information regarding the study’s funding is provided, using the following statement: *“This work was funded by the EQUIACTION funds - https://equiaction.org/ (RP, LL) and IFCE: French Horse and Riding Institute - https://www.ifce.fr/ (RP, LL). These funding sources have no role in the study design, data collection and analyses or in the decision, preparation and submission of the manuscript.”*

3. Please review your reference list to ensure that it is complete and correct. If you have cited papers that have been retracted, please include the rationale for doing so in the manuscript text, or remove these references and replace them with relevant current references. Any changes to the reference list should be mentioned in the rebuttal letter that accompanies your revised manuscript. If you need to cite a retracted article, indicate the article’s retracted status in the References list and also include a citation and full reference for the retraction notice.

All references have been re-added using the PLOS One style via the Zotero software and have been verified. None of the articles have been retracted; however, one of the references that was under revision has been accepted and is therefore mentioned as ‘in press’ in the References section: *‘Lewis, K., McBride, S.D., Micheletta, J., Parker, M.O., Rincon, A.V., Wathan, J. & Proops, L. (in press). An ethogram of facial behaviour in domestic horses: evolutionary perspectives on form and function. PeerJ’*. If this poses any issue, we can easily remove the citation.

Reviewers' comments:

5. Review Comments to the Author

Please use the space provided to explain your answers to the questions above. You may also include additional comments for the author, including concerns about dual publication, research ethics, or publication ethics. (Please upload your review as an attachment if it exceeds 20,000 characters)

Reviewer #1: This is an interesting study looking to assess indicators of positive and negative anticipation in horses. The study is well written and very clear to follow. The idea of regarding multiple behavioural signals at the same time is highly applicable as most horse handlers already do this (often unconsciously). Most of my comments are editorial or to provide more detail in the methodology.

L131 – can you include some justification of why going to the pasture would be perceived as positive?

We mentioned in the Materials and Methods section that the pasture was inaccessible during the period of the study (L169-L171), which highlights the positive impact it would have on the horses.

We include additional justification for why going to the pasture would be perceived as positive for the horses: *'The pasture is known to have a positive impact on horses [55,56] and represented a familiar, highly valued environment, particularly as the individuals did not have access to it during the test period'* (L215-L217).

Moreover, we have slightly modified the wording of the objectives to soften the assertion that the situations would generate positive and negative states, replacing 'was perceived' with 'expected to be perceived': *'One procedure involved the potential for access to a pasture, which was expected to be perceived as a positive stimulus, while the other involved social isolation in a box with predator sounds and new objects, which was expected to be perceived as a negative stimulus'* (L128-L131).

L191 and L202 – it is helpful if figure captions contain all information necessary to understand the figure without the reader having to consult the text. Thus you could include information about what you are testing, what species and how many you are testing, etc. This would apply to all figures and tables in the manuscript.

We have modified all the captions of the tables and figures, including the requested details where appropriate.

L206 – can you clarify what you mean by 'attached with a halter' does this mean the horse was simply wearing a halter or the horse was tethered in to the halter while in the box?

We have modified the sentence to clarify that the horse is tied with two lead ropes in the box and is not standing freely (L208-L209): *'This consisted of placing the individual in a starting box, secured on both sides of the halter with lead ropes, and facing a closed door'.*

L211 – ah, I see now you remove the 'lunges' from the halter, so it would appear the horse was tethered. Better wording would be '…the experimenter untethered the horse to all them….'

Exactly, we've replaced the word 'removed' with 'untethered' as you suggest to make it clearer (L213). We have also replaced the word 'lunges' throughout the manuscript with 'lead ropes', which seems more appropriate.

L214 – can you provide a reason why the experimenter was wearing a green coat? Same on L222 – why a blue coat?

To strengthen the association of the situation with multiple factors, we decided to implement this colour-coding scheme in addition to the location of the experimental box. We added this reason: *'To strengthen the association of the situation with a permanent cue, the experimenter opening the door was always dressed in a green coat' (L219-L220)* and '*To strengthen the association of the situation with a permanent cue, the experimenter opening the door was always dressed in a blue coat': (L229-L230).*

L216 – clarify what you mean by 'the device'. Also L223

We have replaced the word 'device' by 'experimental set-up' (L222 and L232) to be clearer and consistent with the term already used in the caption to Fig 2.

L218 - clarify what you mean by 'guided'. Was the horse led with a lead rope or by the halter?

We clarified that it was about guiding the individual with a lead rope: *'the experimenter guided her with a lead rope towards the pasture*' (L223-L224) and *'the experimenter guided her with a lead rope to the isolation box'* (L233-234).

L219 – how much concentrate was the horse given and was it a feedstuff they were familiar with?

We specified that it was three handfuls of palatable concentrate, indicating that the horses rarely received this type of feed, making it a special event: *'the individual was given three handfuls of palatable concentrate feed'* (L225).

L220 – the isolation box would only be unknown to the horses the first time they experienced it. After that it would be a familiar space.

We agree with this comment and have therefore amended the sentence: *'the corridor led to a box that was initially unfamiliar to the individuals'* (L227-L228).

L227 – what types of objects and how many were in the isolation box? Did they change with each time the horse entered the box? What was the decibel level of the sound recording?

We specified the number of objects and the fact that the tunnel changed position: *'five plastic bags hanging from the walls and one plastic tunnel, which changed position from day to day'* (L236-L237). We also specified the decibel level: *'The sound level ranged between 60 and 75 decibels, depending on the horse's position, as she was free to move around in the isolation box.'* (L238-L239).

L275 – what was the criteria to determine an artifact

We followed the method mentioned by Von Borell E et al., 2007, which specifies that artifacts may result from poor electrode-skin conductance or equipment malfunction and can be seen through visual inspection. The criterion we used to identify an artifact was any isolated point creating a peak that could not be physiological in the heart rate curve, as it represented a change that was too rapid to be biologically plausible. We have added this specification: *'After data collection, heart rate data was visually corrected in order to remove artifacts that might result from poor electrode-skin conductance or equipment malfunction and were detected visually, with the criterion being any isolated point creating a peak too rapid to be physiological in the heart rate curve'* (L286-L289).

L282 – please include some details on how the saliva samples were collected

We have added a sentence specifying how the saliva sample was taken*: 'A cotton swab held by a metal clamp was kept in the horse's mouth between 30 and 40 seconds, positioned above and below the tongue before being placed in Salivettes'* (L299-L301).

L287 – clarify that it was the supernatant that was place in the 1ml tubes

Indeed, we clarify that it was the supernatant: *'then supernatant was placed in 1mL micro-tubes before being frozen at -20°C'* (L304-L305).

L290 – please include information about the immunoassay (supplier, manufacturer). Also did you average the cortisol concentrations from the two 50ul aliquots for each sample?

The immunoassays used were developed at the INRAE Laboratory in Nouzilly, so they do not have specific manufacturers. We have provided more details regarding the techniques used and have confirmed that we calculated the average cortisol concentration of the aliquots: *'The concentration of cortisol in saliva was determined using enzyme immunoassay (EIA), which involved a competition between the cortisol in the saliva and a cortisol alkaline phosphatase solution. Cortisol was measured in two 50 µL aliquots, and all samples* were *analysed in duplicate within the same assay. The limit of detection of the assay was 2 ng/mL. The mean value of cortisol concentration from the two 50 µL aliquots was retained. At the end of the study, eight samples had been collected from each individual (Fig 1)'* (L306-L313).

L316 – is there a reference for the EquiFACS manual?

We have removed the term 'manual' (L343 and L362), as it does not refer to anything new but rather to the methodological article EquiFACS by Wathan et al. (2015), which has already been cited previously (L335-L336).

In Table 1 under Supplementary Behaviours, the first row is observe congener/experiment. Perhaps conspecific is a better word than congener?

We agree and have changed the term 'congener' to 'conspecific' in Table 1.

L427 – here and for all results, it would be appropriate to include the actual statistical values (e.g. F-statistic) along with the p values in the written results. However since you have all the statistical values in Table 2, no need to include them in the text at all.

As we use the *glmmTMB* package to fit our models, the appropriate statistical value is the Z statistic, instead of the F statistic (which is provided by the lme4 package). Although both packages are used to assess the significance of effects, they are based on different statistical methods (Wald test versus ANOVA), both of which are valid. Thus, all necessary statistical values have been provided in the Tables.

L476 – here you report on medium neck position, but L409 indicated that you were only analyzing high and low neck positions.

The two types of analysis (network-based L419 and model-based L404) do not account for the neck position variable in the same way.

The network analysis (L419) quantifies event co-occurrences, with action units (AUs) representing muscle activity. The horse's basal state was defined by the absence of facial movements and a mid-neck position, while networks incorporated AUs and neck position changes (high or low) as events.

However, the model-based analysis (L404) considers neck positions as durations rather than co-occurring events, allowing us to assess whether horses spend more time in a given position depending on the condition.

For clarity, in the Statistical Analysis section of the model-based analysis, we have added a reference to Table 1, which describes the three neck positions in terms of duration (L406).

We also clarify this point in the network analysis with the following sentence: *'The 'Medium Neck Position 'was considered the baseline position; therefore, the two selected neck positions were the 'High Neck Position' and the 'Low Neck Position', as they provide more detailed information about horse's arousal state and were considered as events' (L434-L437).*

L550 – change the word 'equine' to 'equids'

Thank you for pointing this out; we have changed the wording (L593).

L577 – the question of valence vs arousal is a really important point!

Indeed, this is very interesting, and we also find it to be an important point!

L587 – neck position could also be influenced by what the horse is looking at, specifically if it is something up close/far away or above or below their line of vision. Horses must raise their head to be able to focus on an object.

The comment is relevant, and we have added it to the paragraph you have indicated*: ‘However, the position of the neck can also be influenced by what the horse is observing, as horses need to raise their heads to focus, depending on whether it is near or distant, or located above or below their line of sight’* while also adding a reference at the end of the sentence (L630-L632).

L611 – please include a reference for the sentence on the purpose of NetFACS

We included the reference for the sentence (L655).

L659 – I would leave it to the discretion of the editor but generally figures and tables should not appear in the discussion. Figure 5 might instead be very useful as a graphical abstract.

We agree and have therefore incorporated Table 5 and Figure 5 into the Results section: *‘By combining the results of our two analyses, the individual analysis and the network analysis (Table 5), we propose two different facial expression profiles related to positive and negative anticipation (Fig 5)’* (L541-L543).

A careful review of grammar would be helpful. In some cases there is a switch between present and past tense, or word order or choice makes meanings unclear.

A thorough revision has been conducted to ensure consistency in grammar, verb tenses, and terminology, thereby enhancing the clarity of the text. Some minor changes have been made to make the manuscript clearer:

In the Materials and methods and Results sections, see:

- L151: ‘authorisation’ instead of ‘authorization’
- L163: ‘under the’ added to ‘negative condition’
- L230 and L276: ‘untied’ instead of ‘detached’
- L237: ‘recordings of predator calls’ instead of ‘predator call recording’
- L259: ‘had to’ instead of ‘must’
- L265: ‘subjected’ instead of ‘submitted’
- L282: ‘areas’ instead of ‘regions’
- In Table 3: ‘Variable’ instead of ‘Data Type’
- L405, L451, L496: ‘conditions’ instead of ‘condition’

The main corrections were made in the Discussion section to clearly distinguish between the first part, where the past tense is used to validate the positive versus negative condition, and the second part, which includes both past and present tenses to draw broader conclusions on behaviours and facial expressions.

For the first part of the Discussion, see:

- L580: ‘Thus, horses, when given the choice…’ instead of ‘This indicates that horses’
- L584: ‘Arguably’ instead of ‘This can be attributed to the fact that’
- L584: ‘decreased’ instead of ‘decrease’

- L589: ‘Thus’ instead of ‘It can thus be assumed’
- L590: ‘appeared’ instead of ‘appear’
- L591: ‘result’ instead of ‘finding’
- L591-L593: correction of the sentence to be grammatically correct: *‘this result suggests that horses experienced a negative valence in the negative condition, supported by previous reports’*
- L601: ‘were contrasted’ instead of ‘are contrasting’
- L602-L603: correction of the sentence to be grammatically correct: *‘supported by higher cortisol levels in response to negative valence situations observed in horses’*
- L607: ‘validated’ instead of ‘demonstrate’
- L611: ‘anticipations’ instead of ‘anticipation’

For the second part of the Discussion, see:

- L615-L616: ‘were more frequently expressed in’ instead of ‘seemed to characterise’
- L618: ‘have also been observed as’ instead of ‘may also be’
- L626 and L627: ‘reported’ instead of ‘report’
- L635: ‘showed’ instead of ‘show’
- L639: ‘revealed’ instead of ‘reveal’
- L640: ‘that had not been’ instead of ‘that have not been’
- L643: ‘had’ instead of ‘has’
- L653-L655: Fusion of two sentences into one with the addition of a comma
- L661: ‘Through the integration of the two levels of analysis (Table 5)’ instead of ‘Thus’
- L675: ‘was’ instead of ‘is’
- L676-L677: ‘has been identified in the vigilance posture’ instead of ‘is similar to the vigilance posture’
- L679: ‘was’ instead of ‘is’
- L680: ‘which’ instead of ‘that’
- L685: ‘was’ instead of ‘is’

Thanks to these minor corrections, we believe that the text is even more comprehensible than before.

Check references carefully. Some are lacking some information (eg. Ref 17, 18, 46, 59, 61, 67). Many are missing DOIs

We apologise for these shortcomings, which were due to an outdated version of the Mendeley software. All references have been re-added using the PLOS One style via the Zotero software and have been verified.

We followed the guidelines provided by *PLOS One*, which specify the use of the PLOS ONE style (https://journals.plos.org/plosone/s/submission-guidelines#loc-references). In some examples (see the example given: Hou WR et al., 2011), the DOI does not always appear. This is normal, as the DOI is included only when the journal issue and page numbers are not available (e.g., for an online journal). Below is the note provided by *PLOS One*:

“Note: A DOI number for the full-text article is acceptable as an alternative to or in addition to traditional volume and page numbers. When providing a DOI, adhere to the format in the example above with both the label and full DOI included at the end of the reference (doi: 10.1016/j.molimm.2014.11.005). Do not provide a shortened DOI or the URL.”

Reviewer #2: I wish to complement the authors on a well written and organized manuscript. This study is of interest and importance to the field of equine behavior and welfare and provides a novel approach in combination with previously tested/recommended behavioral and physiological indicators to gain further insight into equine affective state. Overall, the methods and results are presented clearly and provide useful information which will aid other researchers in designing studies to further investigate equine emotion for the purpose of assessing welfare and assuring horses are experiencing positive welfare in different management schemes and handling situations.

Specific recommended edits:

Line 122 - Change "methods" to "method"

Thanks for pointing it out, it's been corrected (L120).

In the Materials and Methods section of the manuscript, horses were randomly assigned to groups (i.e., randomly assigned to order in which they would be tested in the positive and negative conditions), correct? It would be helpful to the reader if some additional detail concerning the study horses' routine/previous access to turnout and whether a companion horse is or was typically present during turnout is included.

We have included some details of the horses' routine regarding their access to the paddock which specifies: *'They lived in groups in indoor stalls bedded with straw and had direct, unrestricted access to an outdoor paddock without grass. During the study, which took place between the end of March and early February, the horses did not have access to pasture, as they were not turned out to graze by the caretakers during this period'* (L167-L171).

Lines 206-210 - The term "lunges" is not familiar in this context. Do you mean lead line? Specifically, it isn't completely clear in this statement whether horses are free-standing in the stall or tied in the stall with a halter and lead line/lead rope.

We agree and have replaced 'lunges' with 'lead ropes' throughout the manuscript. We have also modified the sentence to clarify that the horse is tied with two lead ropes in the stall and is not standing freely: *'This consisted of placing the individual in a starting box, secured on both sides of the halter with lead ropes, and facing a closed door'* (L208-L209).

Lines 216 and 218 and later in Line 225 - Study subjects are described as Welsh mares, but here in the methods, the terms "he" and "him" are used when referring to an individual horse.

Indeed, since it refers to mares, we changed 'he' to 'she' and 'him' to 'her' throughout the manuscript.

Line 331 - Change "defines" to "defined"

Thanks for pointing it out, it's been corrected (L355).
